# Severe clinical manifestation of mitochondrial disease due to the m.3243A>T variant: a case report of early-onset, multi-organ involvement and premature death

**DOI:** 10.1007/s44162-025-00110-0

**Published:** 2025-08-08

**Authors:** Hannah Gillespie, Yi Shiau Ng, Katrina M. Wood, Sila Hopton, Charlotte L. Alston, Emma L. Blakely, Nick Thompson, Robert W. Taylor, Andrew C. Browning, Robert McFarland, John A. Sayer

**Affiliations:** 1 Renal Services, Newcastle Upon Tyne Hospitals NHS Foundation Trust, Newcastle Upon Tyne, UK; 2 NHS Highly Specialised Service for Rare Mitochondrial Disorders, Newcastle Upon Tyne Hospitals NHS Foundation Trust, Newcastle Upon Tyne, UK; 3 NIHR Newcastle Biomedical Research Centre, Newcastle Upon Tyne, UK; 4 Directorate of Neuroscience, Royal Victoria Infirmary, Newcastle Upon Tyne Hospitals NHS Foundation Trust, Newcastle Upon Tyne, UK; 5 Department of Cellular Pathology, The Newcastle Upon Tyne Hospitals NHS Foundation Trust, Newcastle Upon Tyne, UK; 6 Mitochondrial Research Group, Faculty of Medical Sciences, Translational and Clinical Research Institute, Newcastle University, Newcastle Upon Tyne, UK; 7 Department of Gastroenterology, Newcastle Upon Tyne Hospitals NHS Foundation Trust, Newcastle Upon Tyne, UK; 8 Newcastle Eye Centre, Royal Victoria Infirmary, Newcastle Upon Tyne, UK; 9 Biosciences Institute, Faculty of Medical Sciences, Newcastle University, Newcastle Upon Tyne, UK

**Keywords:** Mitochondrial disorders, Nephrotic syndrome, MELAS, mtDNA, Membranoproliferative glomerulonephritis

## Abstract

The spectrum of disease associated with pathogenic mitochondrial DNA (mtDNA) variants is wide. Most often, heteroplasmic mitochondrial DNA disease is the result of an adenine to guanine transition at position 3243 of mtDNA (m.3243A > G) in the *MT-TL1* gene encoding tRNA^Leu(UUR)^. Here, we present a case of a patient with a rarer m.3243A > T variant whose phenotype was severe and included delayed growth, developmental delay, myoclonic jerks and tonic–clonic seizures, progressive myopathy, cerebellar ataxia, severe malnutrition due to intestinal dysmotility despite naso-jejunal feeding requiring total parenteral nutrition, bilateral sensorineural hearing loss, and visual impairment, including bilateral cataracts requiring treatment and pigmentary retinopathy. At age 18 years, he developed severe nephrotic syndrome secondary to a membranoproliferative pattern of glomerular injury, which was resistant to treatment and led to premature death.

## Introduction

The spectrum of disease associated with pathogenic mitochondrial DNA (mtDNA) variants is extremely wide [1]. The single most common cause of mitochondrial disease is an adenine to guanine transition at position 3243 of mtDNA (m.3243A > G) in the *MT-TL1* gene encoding tRNA^Leu(UUR)^ [2]. First described in 1990 [3], this has been estimated to account for 80% of cases of mitochondrial encephalomyopathy, lactic acidosis, and stroke-like episodes (MELAS) syndrome [2, 4]. This pathogenic mtDNA variant disrupts mitochondrial protein synthesis, via several proposed mechanisms, leading to defects in oxidative phosphorylation. As a result, tissues with high-energy demands, such as brain, muscle, and heart, experience significant energy deficits, with a spectrum of clinical phenotypes. A less common m.3243A > T variant affecting the same nucleotide in *MT-TL1* has also previously been described in the literature in association with encephalopathy, lactic acidosis, chronic progressive external ophthalmoplegia, and sensorineural hearing loss [5–7], and aminoacylation studies and segregation in single muscle fibres have confirmed its pathogenicity [7].

Mitochondrial diseases have a wide range of phenotypes, often presenting with multi-organ involvement; severity and range of symptoms vary widely amongst affected individuals, in part due to the heteroplasmic nature of pathogenic mitochondrial DNA variants, where the proportion of mutated mtDNA influences the clinical phenotype. Given the abundance of mitochondria within kidney tissues, some mitochondrial diseases have renal phenotyes [8]. Proximal tubulopathy (also known as renal Fanconi syndrome [9]) is the most common renal phenotype in mitochondrial disease, but in addition to tubular injury, all regions of the glomerulus can be affected [10]. Like other inherited mitochondrial disorders, m.3243A > G is associated with a wide range of primary renal manifestations [11–13]. Most often, the phenotypes have been of either renal Fanconi syndrome or focal segmental glomerulosclerosis (FSGS), but the precise incidence of each is unknown [14].

Here, we present a case with a heteroplasmic m.3243A > T variant whose multisystem phenotype included gut failure and treatment-resistant severe nephrotic syndrome secondary to membranoproliferative pattern of glomerular injury and early fatality at 18 years of age.

## Case report

We present the case of a young male, who following an uncomplicated pregnancy, was delivered via caesarean section at term. Apart from a brief admission with neonatal jaundice, he had an unremarkable infancy. Throughout childhood, he had faltering growth. He had been assessed in paediatrics for a potential developmental co-ordination disorder and possible autistic spectrum disorder.

He was first referred to tertiary hospital services by gastroenterology with recurrent vomiting, weight loss, and abdominal pain at 9 years old. A gastric emptying study demonstrated delayed gastrointestinal transit. An oesophagogastroduodenoscopy (OGD) showed oesophagitis with a normal stomach and duodenum. Oesophageal biopsies showed an eosinophil-rich oesophagitis; however, a repeat test showed more typical features of reflux oesophagitis, which was treated with stomach acid suppression.

He developed progressive nutritional failure with a weight less than the first centile. Delayed gastric emptying was confirmed by gastric emptying and barium studies. Enteral nutrition was attempted with nasogastric and naso-jejunal feeding; however, this was not successful due to pain and vomiting, and so parenteral nutrition was commenced and continued thereafter, with his parents providing necessary line care. Subsequently, a gastrostomy tube was placed to allow delivery of medications and gastric drainage to aid management of vomiting.

**Fig. 1:**
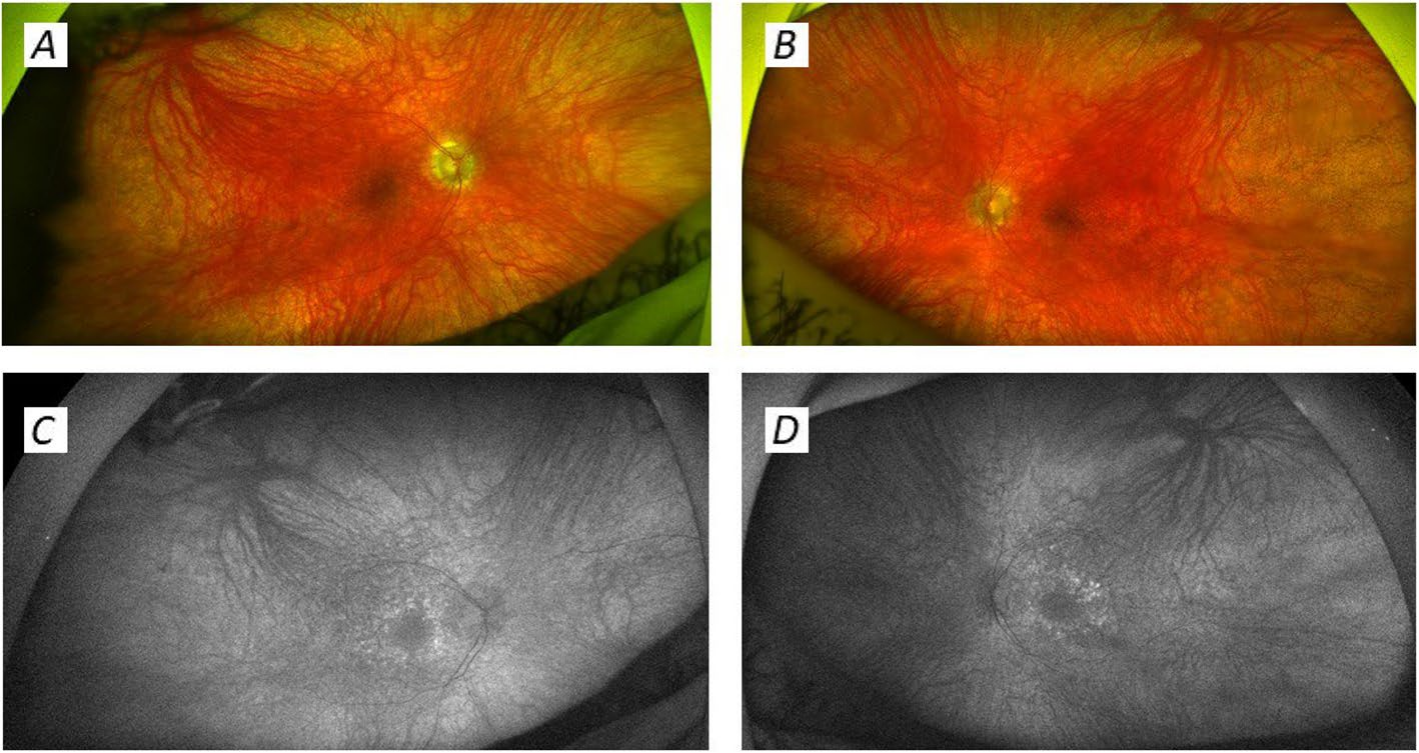
Wide angle fundus colour (**A**, **B**) and autofluorescence images of the patient at age 15. Images **A** (right) and **B** (left) demonstrate a widespread pigmentary retinopathy and retinal vessel attenuation, overlying general retinal RPE pallor. Autofluorescence images **C** (right) and **D** (left) demonstrate bilateral paramacular hyperautofluorescence rings, suggesting the presence of an additional maculopathy

At 13 years of age, he was noted to have static linear growth: now at the 2nd centile for height and 9th centile for weight. At 15 years of age, he developed problems with his hearing and vision and was diagnosed with a bilateral sensorineural hearing loss and visual impairment. He had symptomatic bilateral cataracts requiring intervention, with additional pigmentary maculopathy and retinopathy (Fig. 1). After bilateral cataract surgery, his visual acuity was 6/12 in both eyes. A full-field electroretinogram (ffERG) demonstrated barely detectable rod and cone function, and a macular OCT scan showed generalised macular thinning. He was noted to have an elevated serum lactate (5 mmol/L). He developed myoclonic jerks. At this point, a referral to the mitochondrial service was made given the clinical suspicion of an inherited mitochondrial disease. Alongside this, his academic attainment was noted to decline. He was under regular review from endocrinology, with testosterone started aged 15 years, due to lack of pubertal onset.

At 16 years old, he had his first generalised tonic–clonic seizure. An electroencephalogram (EEG) was performed and revealed changes in keeping with mild encephalopathy. There were also frequent generalised high-amplitude polyspikes and slow wave activity at 1–2-Hz recording. A photic stimulation protocol was attempted. At 1 Hz, with eyes open, the initial flash provoked a large visual evoked potential, and the second and third flash stimuli provoked generalised polyspike and slow wave bursts. These were associated with what appeared to be a generalised myoclonic jerk. Taken together, this showed evidence of encephalopathy, with underlying seizure predisposition and photosensitivity, with apparent stimulus-evoked myoclonus. His MRI head scan did not identify any evidence of subacute cortical signal abnormalities. Levetiracetam was commenced at an initial dose of 200 mg BD, with ongoing up-titration to a final dose of 1000 mg BD.

**Fig. 2:**
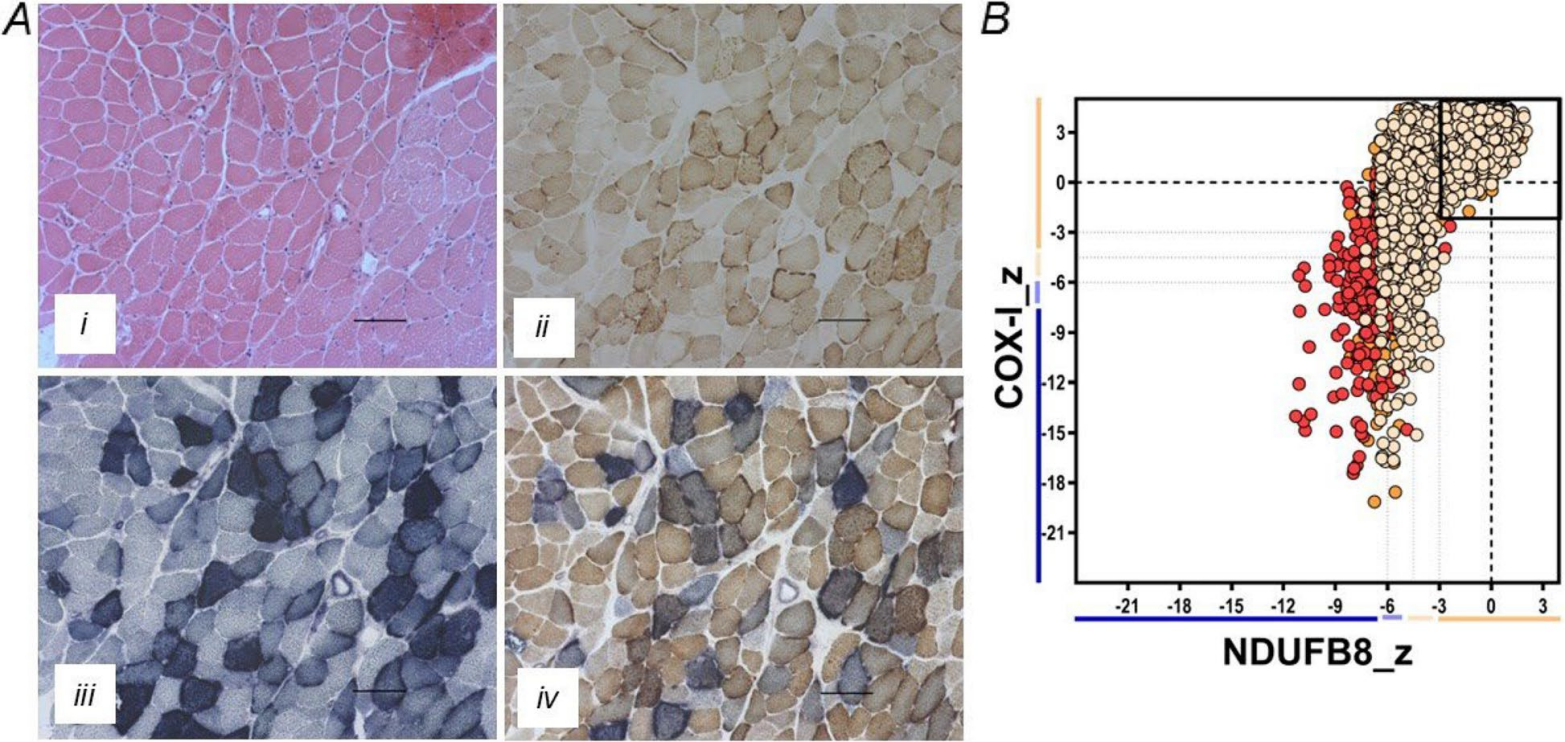
Histopathological studies and assessment of mitochondrial protein levels in patient muscle.** A** Hematoxylin and eosin (H&E) staining (i), cytochrome *c* oxidase (COX) histochemistry (ii), succinate dehydrogenase (SDH) histochemistry (iii), and sequential COX-SDH histochemistry (iv) demonstrate a clear mosaic pattern of COX deficiency, with many fibres showing abnormal, subsarcolemmal accumulation of mitochondria; COX-positive ragged-red fibres are also evident. Scale bar = 100 µm. **B** Quadruple immunofluorescence analysis of NDUFB8 (complex I) and COXI (complex IV). Each dot represents the measurement from an individual muscle fibre, colour co-ordinated according to its mitochondrial mass (low, blue; normal, beige; high, orange; very high, red). Grey dashed lines represent SD limits for classification of the fibres. Lines next to the *x*- and *y*-axis represent the levels of NDUFB8 and COXI: beige, normal (> − 3); light beige, intermediate positive (− 3 to − 4.5); light purple, intermediate negative (− 4.5 to − 6); purple, deficient (< − 6). Bold dashed lines represent the mean expression level of normal fibres. These data nicely confirm a loss of NDUFB8 and COX1 subunit expression in many fibres, consistent with a generalised defect in mitochondrial translation due to the m.3243A > T variant

**Fig. 3:**
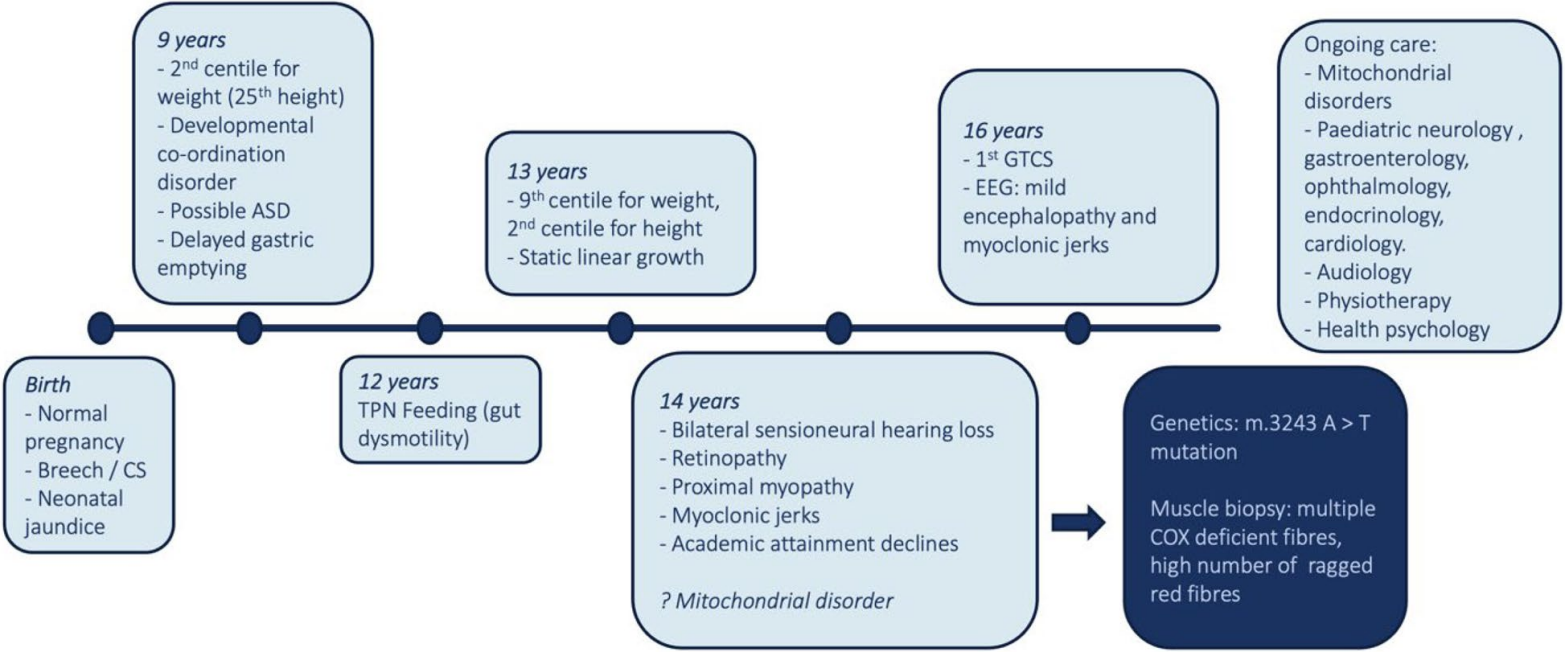
Timeline of patient’s diagnostic journey and clinical phenotypes

At this stage, genetic testing was undertaken. Molecular genetic analysis of the mitochondrial genome in blood revealed the m.3243A > T (NC_012920.1) *MT-TL1* pathogenic variant at intermediate levels of mtDNA heteroplasmy in blood (45%) and fibroblast (61%) DNA samples. A vastus lateralis muscle biopsy was taken from the patient, revealing evidence of atrophic fibres with ill-defined aggregates in places. Numerous fibres showed a basophilic degenerate appearance, with irregularities in the sarcoplasmic reticulum characteristic of ragged-red fibres. Histopathological analyses, specifically oxidative enzyme histochemistry, showed a significant number of COX-deficient muscle fibres which were SDH positive, affecting ~ 15% of the total number of fibres in the biopsy; many fibres showed abnormal subsarcolemmal mitochondrial accumulation (ragged-red fibres), some of which were COX positive (Fig. 2A). Quadruple fluorescent immunohistochemistry of patient muscle confirmed a mitochondrial biochemical defect involving both complexes I and IV, with many fibres exhibiting a significant loss of NDUFB8 and COXI protein expression (Fig. 2B). The *MT-TL1* pathogenic variant was present at 82% in skeletal muscle and at 83% in renal tissue. Figure 3 illustrates the timeline to diagnosis.

Quantitative pyrosequencing of DNA samples obtained from the patient’s mother confirmed maternal transmission of the variant (6% in blood, 14% in buccal epithelia, and 26% in urinary sediment). She started to develop hearing deficit in late childhood and was formally diagnosed with sensorineural hearing loss in her late teens. She had bilateral cataract surgery performed at the age of 40 and developed nyctalopia at age 47. She was diagnosed with a widespread pigmentary retinopathy with additional areas of macular geographic atrophy at the age of 52. Retinal electrophysiology was performed and incorporated the standards of the International Society for Clinical Electrophysiology of Vision (ISCEV) using silver thread electrodes. The full-field electroretinogram (ERG) demonstrated findings consistent with a rod-cone dystrophy, with rod function being barely detectable. The pattern ERG demonstrated moderate macular dysfunction. Overall, the full-field ERG changes, seen in both the proband and his mother, are not typical of the molecularly similar, but more common, m.3243A > G mutation [15]. She experiences exercise intolerance and has slowly progressive muscle weakness. She does not have diabetes mellitus and renal or cardiac involvement. The patient had a younger sister who is fit and well at the age of 17 years. There is no other relevant neurological or early-onset multisystem disease running in the extended family.

His neurological examination at age 17 years showed proximal muscle weakness in limb girdles (MRC grade 4/5) and mild axial weakness with neck flexion and extension (MRC grade 4 +/5). All deep tendon reflexes were present and symmetrical, and the plantar response was downgoing bilaterally. There were signs of mild cerebellar ataxia, including upper limb dysmetria, dysdiadokokinesia, heel-shin incoordination, and an inability to perform heel-toe walking. There was no eyelid ptosis, external ophthalmoplegia, facial weakness, or dysarthria.

At age 18 years, he was referred to Adult Nephrology with a 3-month history of leg and facial swelling, with associated proteinuria. He had frequent headaches and was more lethargic than usual. At the time of referral to Adult Nephrology, his regular medications included the following: esomeprazole 30 mg IV OD, levetiracetam 1 g BD, octreotide 40 mcg IV BD, vitamin D, and IM testosterone.

On review at clinic, he was hypertensive (133/98 mmHg). Blood tests showed hypoalbuminemia (21 g/L, normal range 35–50 g/L), with preserved secretory renal function (serum creatinine 48 µmol/L, normal range 59–104 µmol/L, eGFR [as estimated by revised Schwartz equation] 100 ml/min/1.73 m^2^). Urine protein was 10 g/L. Kidney ultrasound scan showed structurally normal kidneys, measuring 9.5 cm and 9.8 cm. A renal immunology screen was negative. A renal biopsy was undertaken to define further the aetiology of his nephrotic syndrome.

**Fig. 4:**
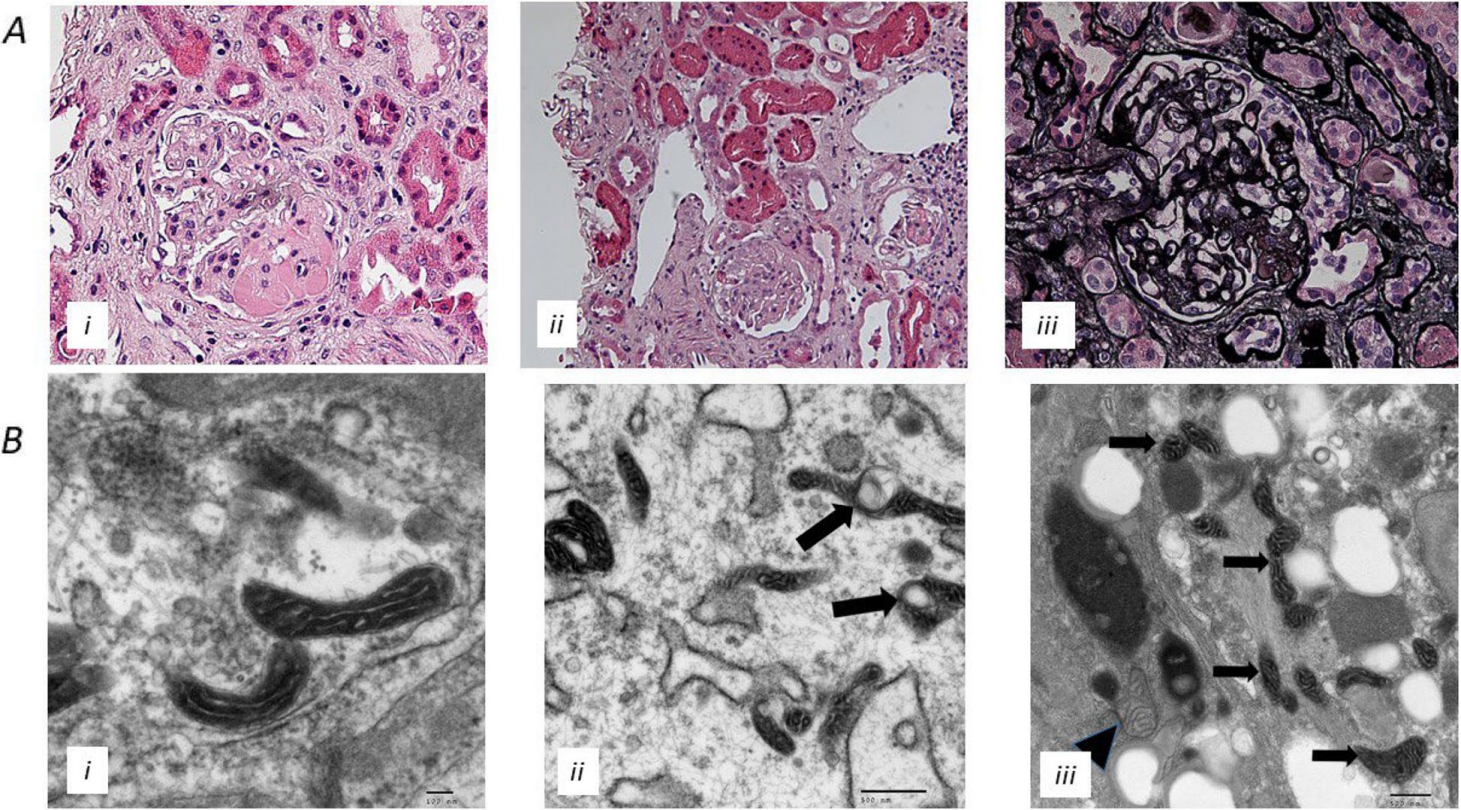
Kidney biopsy following presentation with severe nephrotic syndrome

The findings at kidney biopsy were unusual (Fig. 4). Two cores of renal cortex and medulla were obtained. By light microscopy, three segmental sclerosing/hyalinosis lesions were present within the glomeruli, with moderate chronic tubulointerstitial damage. There was focal segmental trapping of IgM, C3, and C1q on immunofluorescence studies. There was no convincing deposition of IgG, IgA, kappa, or lambda light chains. Electron microscopy of the kidney biopsy identified abnormal mitochondria within the glomerular endothelial cells and podocytes. The overall appearances were consistent with renal involvement from the patient’s known mitochondrial disorder, with focal segmental sclerosing lesions accounting for the patient’s nephrotic syndrome.

For the treatment of his nephrotic syndrome, he was commenced on loop diuretic (furosemide 40–80 mg twice daily) and an angiotensin-converting enzyme inhibitor (lisinopril 10 mg mane, 5 mg nocte), with reasonable effect in improving fluid overload and oedema. His parental nutrition volumes were also adjusted. His hypoalbuminaemia improved slightly, with albumin levels of 26 g/L. He was not systemically anticoagulated or immunosuppressed.

Less than a year after his nephrotic syndrome presentation, he attended a district general hospital generally unwell, with a cough and increasing breathlessness. He was noted to have significant peripheral oedema, and investigation revealed evidence of acute kidney injury (serum creatinine 193 µmol/L, eGFR [as estimated by CKD-EPI equation] 42 ml/min/1.73 m^2^) with worsening hypoalbuminaemia (19 g/L) and a metabolic acidosis (pH 7.26) and elevated lactate (3 mmol/l). White cell count was marginally suppressed (3.8 × 10 > 9/L), but he was not neutropenic. Liver function tests were grossly deranged — ALP 420 (range 20–120 U/L), GGT 740 (range 1–55U/L), with normal bilirubin. At this point, abdominal imaging was repeated, showing normal unobstructed kidneys and a structurally normal liver. Sputum culture had no growth. He was transferred to the tertiary renal centre, where treatment for respiratory infection and oedema was continued with broad-spectrum intravenous antibiotics and intravenous furosemide. Serum creatinine peaked at 277 µmol/L (eGFR [as estimated by CKD-EPI equation] 27 ml/min/1.73 m^2^). Despite treatment, there was a continued generalised clinical deterioration. His repeat neuroimaging showed symmetrical basal ganglia calcification and progressive brain volume loss involving posterior cerebral cortices and cerebellum, without evidence of mitochondrial stroke-like lesion. Both the renal and mitochondrial teams recognised the severity of the current illness in the context of progressive general deterioration in health and were seen by the palliative care team for end-of-life care. He died during this final hospital admission aged 19 years.

## Discussion

This case report presents a severe multisystem disease with an unusual renal manifestation of mitochondrial disease and a rare pathogenic mtDNA variant.

Unlike the more common adenine to guanine transition at position 3243 of mtDNA (m.3243A > G) in the *MT-TL1*, this patient (and his mother) had an adenine to thymine transition (m.3243A > T). The clinical phenotype of six patients with m.3243A > T has previously been reported in the literature [5–7, 16, 17]. Three of these patients have a clinical phenotype of MELAS, one with hearing loss, one with chronic progressive external ophthalmoplegia, and one with rhabdomyolysis [5–7, 16, 17]. Age of onset has ranged from 6 to 29 years [5–7, 16, 17]. One patient died with post-operative acute kidney injury and fluid overload, but aside from this, these cases have not been associated with kidney involvement or nephrotic syndrome. A comparison of these *MT-TL1* m.3243A > T cases is shown in Table 1. Whilst we do not have enough data to ascertain the level of heteroplasmy associated with the A > T variant, we suspect that the subtle phenotype (sensioneural deafness, bilateral cataracts, and a pigmentary retinopathy/macular atrophy) in the mother may be due to higher levels of the variant in postmitotic tissue, as has been reported previously [7].

**Table 1:** Comparison of cases with m.3243A > T variant in *MT-TL1*

Reference	Case	Sex	Onset	Phenotype	Renal involvement
Shaag et al. [5]	1	F	6 y	MELAS	None
Longo et al. [6]	2	F	7 y	MELAS	Postoperative renal failure with fluid overload
Alston et al. [7]	3	M	6 y	Hearing loss	None
Alston et al. [7]	4	F	8 y	Chronic progressive external ophthalmoplegia	None
Czell et al. [17]	5	M	29 y	Rhabdomyolysis	Myoglobinuria
Ikeda et al. [16]	6	M	11 y	MELAS	None
This case	7	M	9 y	As described	Nephrotic syndrome

For patients with mitochondrial disease affecting the kidney, there appears to be a broad spectrum of severity. Previous case reports of the m.3243A > G-related mitochondrial disease have revealed the majority have histological features of FSGS, with abnormal mitochondria evident on EM [18]. These correlate to variable renal manifestations, from mild albuminuria to established nephrotic syndromes [19]. There is limited understanding of the best treatment options for these patients, and like other mitochondrial disorders affecting the kidney, it is unlikely that they will respond to steroid treatment [20]. Furthermore, low muscle mass in some mitochondrial patients means that serum creatinine often underestimates the degree of renal dysfunction, and that eGFR calculations will be inaccurate.

There are several syndromic diagnoses associated with m.3243A > G variants. These include MELAS, maternally inherited diabetes and deafness (MIDD), and chronic progressive exertional ophthalmoplegia (CPEO), but most patients do not fall easily into any of these syndromic diagnoses [21]. It is now recognised that m.3243A > G is associated with a spectrum of clinical features, and patients often develop additional organ involvements with their disease progression following their initial presentation [21]. Diabetes mellitus was included in the initial case description of MELAS syndrome [22] and has been estimated to affect over 40% of adult patients harbouring the m.3243A > G variant [23]. Of note, our patient and his mother, with a m.3243A > T variant, had normal serial HbA1c readings, with no diagnosis of diabetes mellitus.

This case highlights an important practical learning point for the clinical teams who care for patients with mitochondrial disorders. Mitochondrial diseases have a wide range of clinical phenotypes, including kidney disease [11, 24]. Regular monitoring of patients with known mitochondrial diseases for the development of proteinuria (through dipstick testing or urinary protein quantification) and Fanconi phenotypes (such as glycosuria, aminoaciduria, and low-molecular-weight proteinuria) may enable earlier detection and treatment of kidney manifestations before they become otherwise clinically apparent. There is a registry for patients with mitochondrial-related kidney disease (https://www.ukkidney.org/rare-renal/rare-disease-groups/mitochondrial-disease-affecting-kidney).

Given the challenges to obtaining an accurate diagnosis, patients typically present to multiple specialties over the course of their life. There is a further important practical learning point for nephrologists, who routinely assess patients with proteinuria. Obtaining an accurate medical history of other systemic involvement and family history may indicate multisystem involvement and the possibility of a mitochondrial disorder, leading to genetic testing. All clinicians should have a high index of suspicion of mitochondrial disease when facing individual patients presenting with seemingly unrelated multisystem disorders, irrespective of their age, including steroid-resistant nephrotic syndromes [20]. Genetic testing can be performed from urinary sediment, as evidenced by the variant analysis in samples from our patient’s mother, offering a less invasive strategy than sampling postmitotic tissues like muscle; it is particularly useful for monitoring and testing maternally related family members.

For patients with rare genetic diseases, the long, often difficult journey to a precise molecular diagnosis is termed the ‘diagnostic odyssey’. For this patient and his family, diagnosis of a potential mitochondrial disorder took years from his index presentation to gastroenterology with faltering growth and vomiting. The constellation of severe gut dysmotility and elevated blood lactate, alongside a material history of sensorineural hearing loss, raised clinical suspicion of a mtDNA-related disorder and then allowed more precise testing to be performed.

It also shows the true multidisciplinary nature of care for patients with mitochondrial disease manifesting with multisystem complications. Our patient, following initial investigations, was cared for by the NHS Highly Specialised Service for Rare Mitochondrial Disorders, with extensive input from a range of specialists in gastroenterology, endocrinology, nephrology, neurology, ophthalmology, and cardiology, in addition to support from general practitioners and allied health professionals, including audiology, physiotherapy, occupational therapy, and educational and health psychology. The coordination of care between these specialists is no small task and remains a challenge for many rare disease patients.

The patient’s parents shared valuable insights about the lived experience of caring for their son throughout his life, including their reflections on the diagnosis and treatment of his disease. Table 2 includes a full entry from his parents. They reflected that it was important to receive a diagnosis, which for them helped them prove invaluable in steering his care away from unhelpful avenues (for instance, attributing his weight loss to mental health issues) and avoided any further unnecessary treatments. They felt that they lacked a single person who co-ordinated their son’s care, and, instead, this role was one they provided. They suggested that a coordinator would be incredibly valuable to oversee multi-disciplinary care, particularly for the transition period from paediatric to adult services. Finally, they shared that earlier conversations about end-of-life care, with support for the family’s mental health and need for respite support, would have helped them greatly at a time of enormous difficulty.

**Table 2:** A reflection from the patient’s parents

"We hope our son’s experience informs more cohesive, proactive care for future patients and their families"
Diagnosis
It took several years for our son’s genetic diagnosis to arrive, but it proved invaluable in steering his care away from unhelpful avenues (for instance, attributing his weight loss to mental health issues). However, once the diagnosis was established, we felt that certain teams seemed to consider their role had ended in terms of being proactive about further symptom management
Coordinated care
The key missing element in our son’s care was a single person or team to oversee and coordinate all aspects of his treatment. We often felt we were performing that role ourselves. A dedicated coordinator or manager would also have been invaluable when dealing with local hospitals and GP services, which were simply not equipped to handle his complex condition. In his final local admission, incorrect treatment before transfer to tertiary care made his condition considerably worse
Transition to adult services
We felt the transition from paediatric to adult services was very uneven across departments. Almost all teams told us to expect some problems. Why this known issue remained unaddressed in this manner was quite baffling to us. Again, a dedicated coordinator role would have been the ideal candidate to make this whole experience less frustrating
Psychosocial support
The emotional and physical toll on both our son and our family was significant. We did not know how best to help our son understand his condition and what it meant for his life expectancy. While our focus was naturally on his health, we also would have benefited from support to help us cope with the stress and grief over many years of uncertainty. We also had no options for respite during years of providing care and medication for him, particularly during the last years of his life where he was more or less bed bound
Advance care planning
In hindsight, discussions about advanced or palliative care options earlier might have allowed for better-informed decisions and preparation for the inevitable. Instead, his final admission became a series of spiralling crises which we were unprepared for and during which we felt wholly powerless. We feel that a coordinated framework that includes psychosocial and mental health support, counselling, and advance care planning would be hugely beneficial for patients dealing with life-limiting conditions

## Conclusion

This case report presents unusual renal manifestations of mitochondrial disease in a young patient with a rare genetic *MT-TL1* variant. Our patient, who has multisystem disease and nephrotic syndrome secondary to membranoproliferative glomerulonephritis, is one of only a handful of patients with a known m.324A > T variant [17] and only the fourth case in the published literature associated with the clinical phenotype resembling MELAS [5, 6, 16].

We advocate screening for kidney involvement in all patients with mitochondrial DNA disease to allow early detection and treatment of kidney complications. Urinary protein quantification is a cheap and relatively noninvasive test, which should aid early identification of potential kidney involvement. Conversely, nephrologists should remain cognisant of potential genetic causes in patients with renal disease, particularly in those with multisystem disease or for those who do not respond to treatment as expected.

## Data Availability

No datasets were generated or analysed during the current study.
